# Multiple Phosphonate Degradation Pathways in *Amphidinium carterae*-Associated Bacteria Potentially Contribute to Host Adaptation to Phosphorus Limitation

**DOI:** 10.3390/microorganisms14092028

**Published:** 2026-09-11

**Authors:** Yudong Cui, Simin Lu, Dongmei Su, Zhaobin Huang

**Affiliations:** 1 College of Oceanology and Food Sciences, Quanzhou Normal University, Quanzhou 362000, China; 2 Fujian Province Key Laboratory for the Development of Bioactive Material from Marine Algae, Quanzhou Normal University, Quanzhou 362000, China

**Keywords:** phosphonate, dinoflagellate, dinoflagellate-associated bacteria, *phn* genes, phosphonate degradation pathways

## Abstract

Phosphonates in the ocean can serve as an alternative phosphorus (P) source for microorganisms when phosphate is scarce. Dinoflagellates cannot utilize phosphonates, but some associated bacteria can degrade these compounds and release phosphate. However, the community composition of these bacteria and their phosphonate degradation pathways remain poorly understood. In this study, the dinoflagellate *Amphidinium carterae* was cultured with 2-aminoethylphosphonic acid (2-AEP) as the exclusive P source. Metagenomic and genomic analyses were conducted to identify bacterial taxa and genes related to phosphonate utilization (*phn* genes). Specific strains were isolated from 2-AEP cultures to characterize phosphonate degradation gene clusters. Ten of the 20 most abundant genera were found to possess the genetic potential for phosphonate utilization, with *Labrenzia*, *Phycocomes*, and *Pseudosulfitobacter* as the dominant genera. The identified *phn* genes constituted multiple pathways, including the C-P lyase, PhnW-PhnX, and PhnW-PhnY-PhnA pathways, indicating that *A. carterae*-associated bacteria can utilize phosphonates through diverse mechanisms, with some strains even possessing more than one pathway. Five isolated bacterial strains were confirmed to be capable of degrading 2-AEP. These findings underscore the ecological significance of bacterial diversity and metabolic versatility in helping dinoflagellate hosts adapt to P scarcity, providing novel insights into bacteria-algae interactions and marine P cycling.

## 1. Introduction

Phosphorus (P) is an element indispensable for the growth of all living organisms, including marine phytoplankton, which are the most important primary producers in marine ecosystems [1]. Marine waters contain various dissolved P forms, among which dissolved inorganic phosphate (DIP) is the most readily assimilable form for phytoplankton. Nevertheless, DIP is usually present at very low levels in the euphotic zone because of the rapid uptake by phytoplankton, which renders P availability a key limiting factor for marine primary productivity and nitrogen (N) fixation [2,3,4]. Furthermore, intensified water column stratification driven by global warming is expected to further restrict P availability in the surface ocean [5]. Unlike DIP, whose concentration increases gradually with depth, dissolved organic phosphorus (DOP) is accumulated in the surface water, which makes it a potential alternative P source for phytoplankton groups such as diatoms and dinoflagellates [6,7].

DOP in the marine environment mainly exists as phosphoesters and phosphonates, which constitute approximately 75% and 25% of the high-molecular-weight (HMW) DOP pool, respectively [8]. Phosphoesters, such as phosphomonoesters and phosphodiesters, are characterized by C-O-P bonds. Many phosphoesters are important structural and functional components of cells, existing in the form of nucleic acids, phospholipids, and adenosine triphosphate (ATP). These compounds can be hydrolyzed into inorganic phosphate through the action of enzymes such as alkaline phosphatase (AP), phosphodiesterase, and 5′-nucleotidase [7,9,10]. In contrast, phosphonates contain stable C-P bonds, rendering them resistant to degradation. Their metabolism requires specific enzymatic pathways, such as the C-P lyase pathway, the phosphonatase pathway (PhnW-PhnX pathway), and other C-P hydrolytic pathways [11,12]. 2-aminoethylphosphonate (2-AEP) is the most widely distributed phosphonate in marine environments and has been detected in diverse marine organisms, including marine invertebrates, cyanobacteria, dinoflagellates, and coccolithophores [13]. Phosphonates can serve as a component of cell membranes to enhance their structural rigidity and resistance to enzymatic hydrolysis [14]. They are also found to exist in some extracellular polysaccharides and glycoproteins, which are involved in biofilm formation and cell surface modification. Certain phosphonates, such as fosfomycin, can function as secondary metabolites with antimicrobial properties [15].

Recently, most of the research related to the degradation of phosphonates has predominantly focused on bacteria, including cyanobacteria [11,16]. Although certain microalgal species have been reported to have the ability to utilize phosphonates as a P source, most eukaryotic phytoplankton, including dinoflagellates, are not capable of utilizing phosphonates [17,18]. Dinoflagellates are a prominent source of harmful algal blooms (HABs) and also one of the main contributors to primary productivity in the marine ecosystem. Although genes related to phosphonate metabolism were identified in dinoflagellates, they are unable to utilize extracellular 2-AEP, possibly due to the lack of necessary transporters [19]. However, bacteria associated with dinoflagellates can effectively degrade phosphonates, releasing significant amounts of phosphate to meet the P demands of their algal hosts [20]. The growth of *Prorocentrum donghaiense* has been observed to accelerate after 60 days of adaptation when glyphosate, a kind of prevalent anthropogenic phosphonate, was supplied as the only P source, implying that glyphosate had been converted into phosphate by the bacteria associated with this dinoflagellate [21].

Therefore, the dinoflagellate-associated bacteria could be an important mediator for their algal host to acquire P from phosphonates in the marine environment. This mechanism has the potential to facilitate the adaptation of dinoflagellates and even contribute to the outbreak of blooms in phosphate-limited waters. Previous studies have typically treated bacterial communities as a single entity and viewed bacterial degradation of phosphonates as a general concept [19,20,22]. However, further research is necessary to determine which taxonomic groups within the associated bacterial community are responsible for phosphonate degradation, which specific species are involved, and the metabolic pathways they use to utilize phosphonates.

*Amphidinium carterae* (Hulburt, 1957) is a globally distributed dinoflagellate that produces hemolytic toxins and forms HABs [23]. In this study, we investigated the bacterial biodiversity and phosphonate degradation pathways in *A. carterae*-associated bacteria. The algae were cultured under conditions with 2-AEP as the sole P source. Metagenomic analysis was employed to explore the bacterial community composition in the 2-AEP treatments and to identify the genes related to phosphonate utilization (*phn* genes). These genes were assigned to corresponding taxonomic groups to identify the bacterial groups that are potentially capable of degrading phosphonates. In addition, bacterial strains were isolated from the AEP condition, and culture experiments were performed to verify whether they are capable of degrading 2-AEP. This is the first study to systematically explore the dinoflagellate-associated bacterial community related to phosphonate utilization. The results provide novel insights into the diversity of phosphonate degradation pathways in dinoflagellate-associated bacteria and their potential ecological roles in P-limited marine environments.

## 2. Materials and Methods

### 2.1. Algal Cultures and P Treatments

Algal cells of *A. carterae* (CCMP1314) were cultured in an L1 medium at 20 °C and at a light intensity of 100 ± 10 μE·m^−2^·s^−1^ with a photocycle of 12 h light/12 h dark. The medium was prepared using natural seawater from the Western Pacific Ocean. The seawater was first filtered through 0.22-μm membranes, then diluted with deionized water to a final salinity of 28‰, as confirmed by a handheld salinity refractometer. After salinity adjustment, the medium was autoclaved for sterilization. The algal cultures were initially exposed to a treatment of 6 μM phosphate, whose concentration in the standard L1 medium is about 36 μM, until the algal density reached a plateau due to P limitation. The algal cells were then inoculated into three treatments with different P sources: +P condition (36 μM NaH_2_PO_4_), AEP condition (36 μM 2-AEP; Sigma-Aldrich, St. Louis, MO, USA), and -P condition (no P source added). Each treatment was set up with three biological replicates. All cultures were grown in Erlenmeyer flasks containing 300 mL of growth medium, which were placed statically (without shaking) in an incubator that was temperature- and light-controlled. The flasks were gently hand-swirled once a day to resuspend the cells and facilitate gas exchange. No additional CO_2_ was supplied during the cultivation period. In antibiotic-treated cultures, a combination of antibiotics with final concentrations of 100 mg/L ampicillin, 50 mg/L kanamycin, and 50 mg/L streptomycin was added to effectively suppress bacterial degradation of 2-AEP, as confirmed by the loss of algal growth under AEP conditions while +P cultures with the same antibiotics grew normally [19]. Algal cell densities were determined by manual counting using a Sedgwick-Rafter counting chamber (Phycotech, St. Joseph, MI, USA) under a microscope. The maximum quantum yield of PSII (Fv/Fm) of the algal cells was measured using the Water-PAM (Heinz Walz GmbH, Effeltrich, Germany) following a 30 min dark adaptation period at 20 °C. DIP concentrations were determined through the molybdenum blue method [24]. AP activity was measured following the p-nitrophenyl phosphate (pNPP) method as described [25]. Absorbance measurements were performed using an Infinite M200 Pro microplate reader (Tecan, Männedorf, Switzerland).

### 2.2. Filtrate-Based Incubation Experiments

*A. carterae* cells were inoculated into a fresh L1 medium without antibiotics and cultured under the same temperature and light conditions as described above until they reached the exponential growth phase. The algal culture was then filtered through sterile 3-μm membranes to remove algal cells, and the resulting algal cell-free filtrate was collected. The filtrate was dispensed into Erlenmeyer flasks (120 mL per flask) and incubated under the same temperature and light conditions as the algal cultures described above. Two treatments were established: FL condition (filtrate only) and FL+2-AEP condition (filtrate amended with 36 μM 2-AEP). Each treatment was set up with three biological replicates. DIP concentrations were measured every two days throughout the incubation period.

### 2.3. Metagenomic DNA Preparation, Sequencing, and Genome Assembly

Samples were collected from the AEP condition on the 10th day of the algal culture experiment. The algal cultures were first filtered through 3-μm sterile membranes to remove the algal cells, and the filtrates were then filtered through 0.22-μm sterile membranes to obtain the bacterial cells. Metagenomic DNA of the bacterial community was extracted using the Mag-Bind^®^ Soil DNA Kit (Omega Bio-tek, Norcross, GA, USA) following the manufacturer’s protocol.

The DNA was then fragmented to ~350 bp using a Covaris M220 ultrasonicator (Gene Company Limited, Shanghai China). Sequencing libraries were constructed with the NEXTFLEX Rapid DNA-Seq Kit (Bioo Scientific, Austin, TX, USA), during which adapters were ligated. Paired-end sequencing was performed on the Illumina NovaSeq 6000 platform using the S4 Reagent Kit v1.5 (300 cycles) at Majorbio Bio-Pharm Technology Co., Ltd. (Shanghai, China), following the manufacturer’s instructions. Sequence data associated with this project have been deposited in the NCBI Short Read Archive database (Accession Number: SAMN57539599~SAMN57539601).

Metagenomic data analysis was performed online through the Majorbio Cloud Platform (www.majorbio.com). To summarize, Fastp (version 0.20.0; https://github.com/OpenGene/fastp, accessed on 28 July 2024) [26] was used to quality-filter paired-end Illumina reads by trimming adapter sequences and eliminating low-quality reads (length < 50 bp or average quality score < 20). High-quality reads were then de novo assembled using MEGAHIT (version 1.1.2; https://github.com/voutcn/megahit, accessed on 28 July 2024) [27], which applies a succinct de Bruijn graph-based algorithm. Contigs with lengths ≥ 300 bp were retained as the final assembly and subsequently used for gene prediction and functional annotation.

### 2.4. Gene Prediction, Taxonomic Classification, and Functional Annotation

Open reading frames (ORFs) were predicted from assembled contigs using Prodigal (version 2.6.3; https://github.com/hyattpd/Prodigal, accessed on 29 July 2024) [28]. Predicted ORFs with a length ≥ 100 bp were retained and translated into amino acid sequences according to the NCBI genetic code (https://www.ncbi.nlm.nih.gov/Taxonomy/taxonomyhome.html/index.cgi?chapter=cgencodes, accessed on 29 July 2024). A non-redundant gene catalog was generated using CD-HIT [29] (version 4.6.1; http://cd-hit.org, accessed on 29 July 2024) with a sequence identity threshold of 90% and a coverage threshold of 90%. SOAPaligner [30] (version 2.21) was used to align high-quality read sequences against the generated gene catalog (with a minimum homology threshold of 95%) to calculate gene abundance.

Taxonomic annotation and functional annotation of the representative sequences from the non-redundant gene catalog were conducted using DIAMOND (version 2.0.13; https://github.com/bbuchfink/diamond, accessed on 29 July 2024), and the e-value cutoff was set to 1e-5 in both cases [31]. The non-redundant (NR) protein database from NCBI was used as the reference database for the alignment in taxonomic annotation. For functional annotation, three reference databases were used: the NCBI NR database, the eggNOG database, and the KEGG database (http://www.genome.jp/kegg/, accessed on 29 July 2024).

Community pie charts at the phylum, class, and genus levels were generated to visualize the taxonomic composition, with the top 20 most abundant genera displayed and the remaining taxa grouped as “Others.” The *phn* genes were identified from the functional annotation results and then attributed to corresponding genera in order to investigate the bacterial groups that potentially possess the ability to utilize phosphonate.

### 2.5. Isolation and Identification of Pure Bacterial Strains

Two kinds of media were used to isolate the pure bacterial strains from the 2-AEP cultures. One is 2216E marine broth (MB) medium (BD, Cat. 279110, USA), and the other medium was modified from the recipe previously used for cultivating the model marine bacterium *Ruegeria pomeroyi* DSS-3 [32]. The latter was designed as an RP medium in which natural seawater was used to dissolve the macronutrients, trace elements, and vitamins. The liquid media were sterilized at 121 °C for 20 min following their preparation. For solid plates, agar (BD, Cat. 214010, USA) was added at a concentration of 15 g/L. Algal cultures under 2-AEP treatments on the 10th day were collected (500 μL per replicate), pooled, and serially diluted (10^1^–10^6^) with sterile seawater. Diluted samples were spread onto solid agar plates. The inoculated plates were incubated at 30 °C for up to 10 days, and colonies with distinct morphologies were selected and purified by repeated streaking as they became visible at different time points. The purified isolates were then cultured in liquid medium at 30 °C, 180 rpm for 48 h. Genomic DNA was extracted from the bacterial isolates, and the 16S rRNA gene was amplified using universal primers 27F (5′-AGAGTTTGATCCTGGCTCAG-3′) and 1492R (5′-GGTTACCTTGTTACGACTT-3′). The PCR amplicons were then sequenced using the Sanger method. The resulting 16S rRNA gene sequences were compared with reference sequences in the GenBank database using BLAST (https://blast.ncbi.nlm.nih.gov/Blast.cgi, accessed on 10 May 2025) for preliminary species assignment. A neighbor-joining phylogenetic tree of 16S rRNA gene sequences from these bacterial isolates and their close relatives was constructed using MEGA6 software [33].

### 2.6. Genome Sequencing, Annotation, and Gene Identification

Genomic DNA of the bacterial isolate was submitted to Majorbio Bio-Pharm Technology Co., Ltd. for high-throughput sequencing using the Illumina platform. Raw sequencing reads were first quality-filtered using Sickle (https://github.com/najoshi/sickle, accessed on 29 July 2025), and then assembled into contigs using SPAdes [34]. Contigs longer than 1 kb were retained as part of the draft genome for subsequent analysis. The RAST (Rapid Annotation using Subsystem Technology) server was used to perform the annotation of the bacterial genomes [35]. The *phn* genes were identified from the annotation results, and their arrangement in the gene cluster and the pathways they constitute were analyzed. In the analysis of bacterial genomes conducted in this study, the presence of a majority of the genes encoding the C-P lyase pathway, particularly the core genes *phnG*~*phnM*, within a given genome was indicative of the possession of a complete C-P lyase pathway. The presence of both *phnW* and *phnX* is regarded as constituting the PhnW-PhnX pathway. The presence of all three genes (*phnW*, *phnY*, and *phnA*) within a genome indicates the existence of the PhnW-PhnY-PhnA pathway.

### 2.7. Verification of Bacterial Phosphonate Degradation

An RP medium was deployed in this process to establish various P treatments, as it is a synthetic medium in which the P source and its concentration are adjustable. The bacterial strains were initially cultured in a standard RP medium to obtain actively growing cells. The bacterial cultures were then subjected to centrifugation at 10,000 rpm for 10 min to remove the supernatant, and the bacterial pellet was then resuspended in sterile seawater. This procedure was performed twice to effectively remove any P from the previous culture medium. The resulting bacterial suspension was then inoculated into three different P treatments based on RP-P medium. The experimental conditions included +P condition (36 μM NaH_2_PO_4_), AEP condition (36 μM 2-AEP), and -P condition (no P added), with each experimental condition established in triplicate. The OD_600_ was measured to monitor the growth of the bacteria, and DIP concentrations and AP activity were also measured using a similar method applied in the algal experiments. The unit of bacterial AP activity is expressed as mol pNP·OD^−1^·h^−1^, representing the amount of pNP produced per OD of bacteria per hour from the conversion of pNPP.

### 2.8. Statistical Analysis

For the algal and bacterial culture experiments, one-way analysis of variance (ANOVA) was carried out through the PASW Statistics 18 software package to determine significant differences among the various P conditions. Homogeneity of variances was examined using Levene’s test, and Welch’s ANOVA was applied as a robust alternative to account for potential heteroscedasticity. The data were presented as the mean ± standard deviation (SD), and differences were considered significant with the threshold *p*-value of < 0.05.

## 3. Results

### 3.1. Growth Response of A. carterae Under Different Phosphorus Conditions

**Figure 1:**
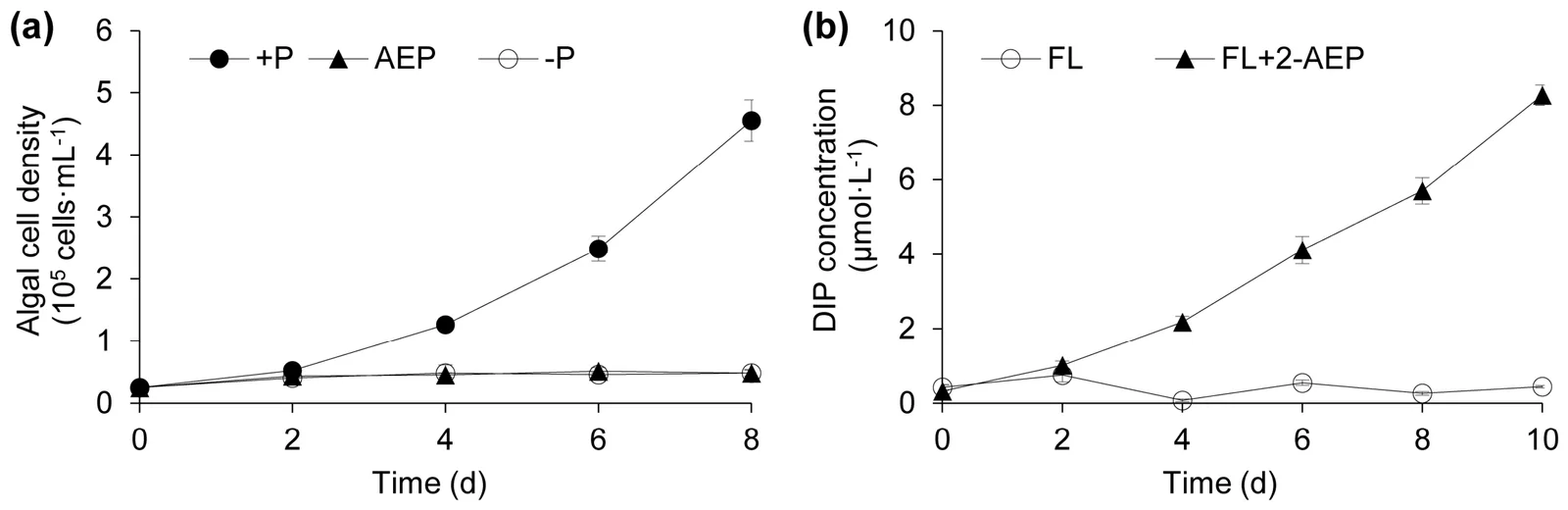
Growth of *Amphidinium carterae* (algal host, bacterial activity minimized) and DIP dynamics in its associated bacterial community (bacteria retained, algae removed) under different phosphorus treatments. (**a**) Cell density of *A. carterae* under different P treatments with antibiotic treatment: +P (36 μM NaH_2_PO_4_), AEP (36 μM 2-aminoethylphosphonate), and -P (no phosphorus added). (**b**) DIP concentrations in algal cell-free filtrate derived from non-antibiotic-treated *A. carterae* cultures, with or without 2-AEP addition: FL (filtrate only), FL+2-AEP (filtrate amended with 36 μM 2-AEP).

**Figure 2:**
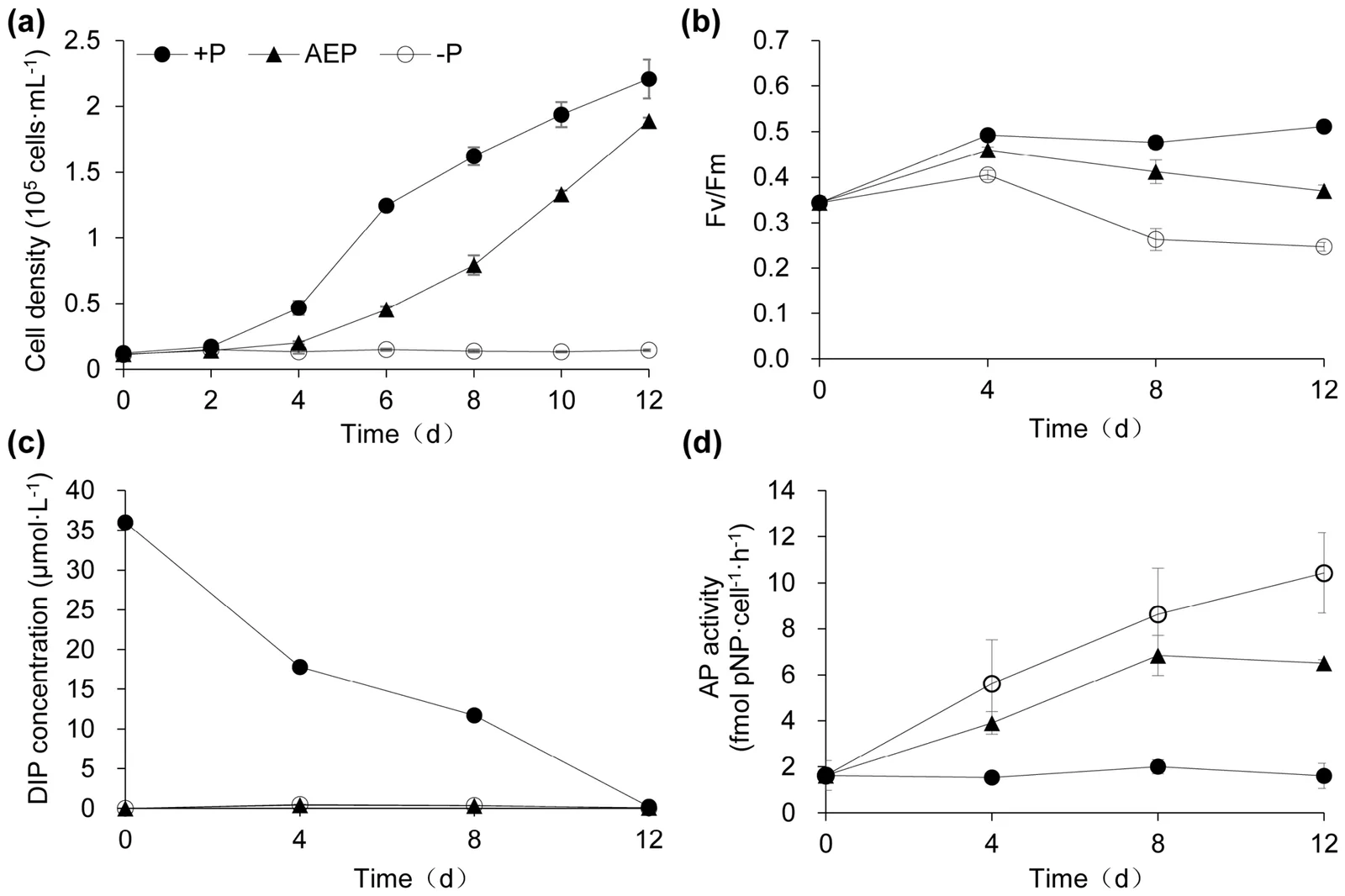
Effects of different phosphorus treatments on the growth and physiological responses of *Amphidinium carterae*. (**a**) Cell density; (**b**) maximum quantum yield of PSII (Fv/Fm); (**c**) dissolved inorganic phosphate (DIP) concentration; (**d**) alkaline phosphatase (AP) activity. Cells were cultured under three phosphorus treatments: +P (36 μM NaH_2_PO_4_), AEP (36 μM 2-aminoethylphosphonate), and -P (no phosphorus added).

*A. carterae* was cultured under three conditions: +P, AEP, and -P, with and without antibiotic treatment. Under antibiotic treatment, algal cell density increased significantly under the +P condition; however, algal cell growth under the AEP condition did not differ from that under the -P condition (Figure 1a). However, under conditions without antibiotic treatment, the growth of *A. carterae* exhibited a distinct pattern (Figure 2). In the +P condition, algal cell densities demonstrated a rapid increase after an initial adaptation period of about 2 days, while the DIP concentration exhibited a rapid decrease over time. The AP activity in this condition was maintained at a low level, while Fv/Fm was maintained at a high level, indicating that the P was sufficient to support the rapid growth of the algal cells in this condition. Conversely, the algal cell density in the -P condition had nearly ceased, and the Fv/Fm increased slightly on the 4th day and then decreased significantly on the 8th and 12th days. The degree of DIP concentration in the -P condition was sustained at a value near zero, while the AP activity demonstrated a continuous increase throughout the 12-day culture period. This indicates that algal cells in the -P condition had experienced a consistent P limitation. For the AEP condition, the algal cell densities showed a slow but consistent increase and were higher than those in the -P condition from the 8th day (*p* < 0.05). The Fv/Fm in the AEP condition was higher than in the -P condition (*p* < 0.05), the AP activity was lower (*p* < 0.05), and the DIP concentration remained at a similarly low level (*p* > 0.05). These results suggest that although algal cells in the AEP condition also experienced P limitation, the extent of limitation was less severe.

### 3.2. DIP Changes in Algal Cell-Free Filtrate Amended with 2-AEP

During the 10-day culture period, the DIP concentration under FL conditions exhibited slight fluctuations but generally remained at a low level (less than 0.75 μM), with no apparent upward trend (Figure 1b). Conversely, DIP concentrations demonstrated a marked increase under FL+2-AEP conditions, reaching levels significantly higher than those observed under FL conditions starting on Day 4 (*p* < 0.05).

### 3.3. Bacterial Community Composition Under AEP Conditions

The sequencing and assembly statistics for the three AEP replicates are summarized in Table S1. Briefly, an average of 45.4 million clean reads (range: 42.8–47.9 million) were obtained per sample, yielding assembled contigs with N50 values ranging from 103.5 to 114.2 Kb and total assembly sizes of 80.9–86.2 Mb per sample.

**Figure 3:**
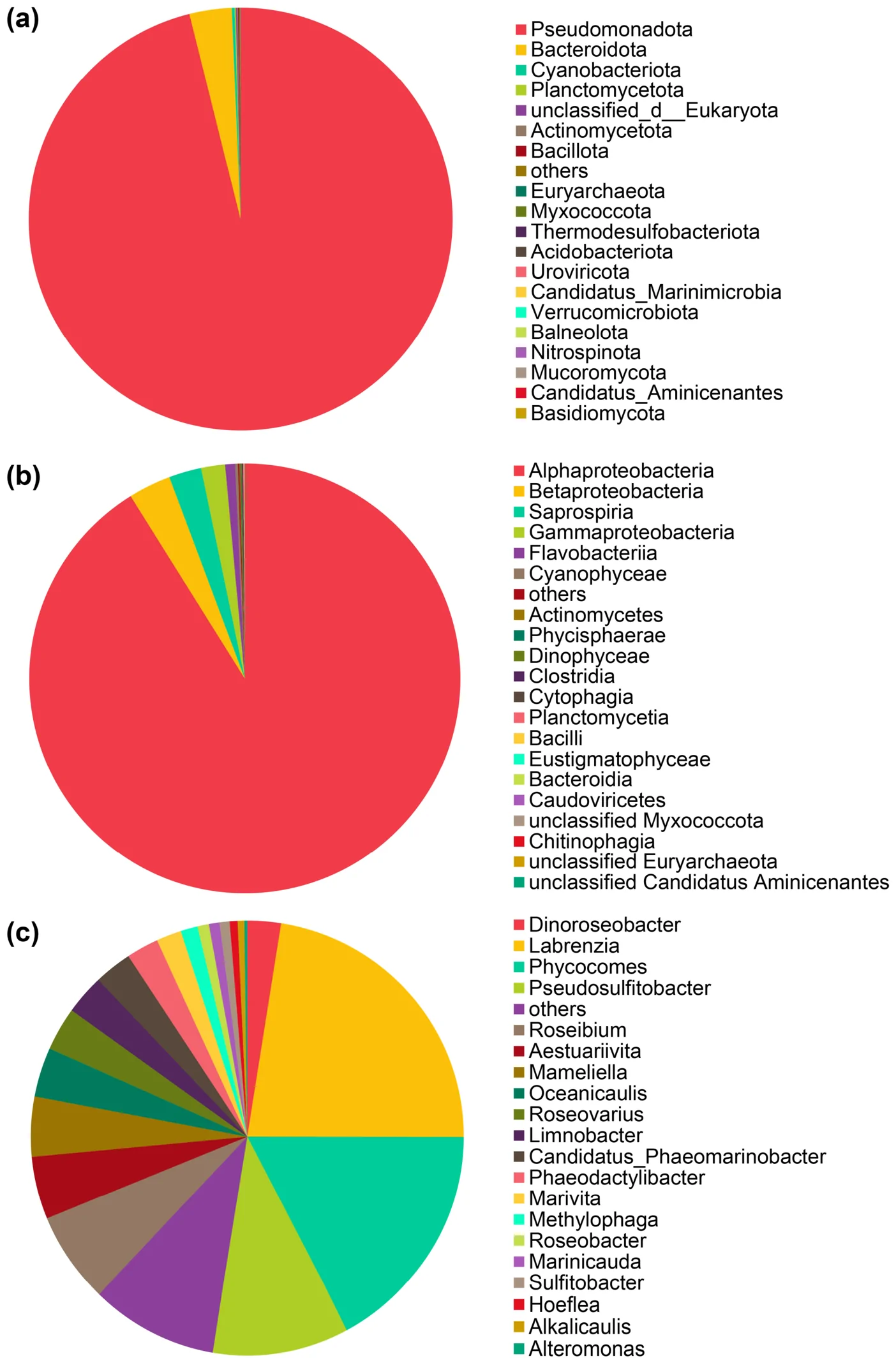
Bacterial community composition under AEP conditions at the phylum (**a**), class (**b**), and genus (**c**) levels.

Metagenomic analysis results indicate that the majority of bacteria under AEP conditions belong to Pseudomonadota, which accounts for 96.1% of the relative abundance and is the dominant phylum (Figure 3a). Bacteroidota is the second most abundant phylum, accounting for 3.22%. The relative abundance of other phyla was less than 1%, with most being less than 0.25%. At the class level, Alphaproteobacteria was the dominant class, comprising 91.1% of the bacterial community (Figure 3b). The proportions of other classes were significantly lower than those of Alphaproteobacteria. Betaproteobacteria (3.19%), Saprospiria (2.43%), and Gammaproteobacteria (1.80%) were the second through fourth most abundant groups.

At the genus level, *Labrenzia* (22.52%), *Phycocomes* (17.38%), and *Pseudosulfitobacter* (10.15%) were the three most abundant genera under AEP conditions, constituting approximately 50% of the community (Figure 3c). From the perspective of the broader taxonomic levels, it was found that 19 of the 20 genera with the highest total abundance across all samples belonged to the Pseudomonadota, with the exception of *Phaeodactylibacter*, which belonged to the phylum Bacteroidota (class Saprospiria). Among the 19 genera in the Pseudomonadota, 16 belong to the Alphaproteobacteria, one to the Betaproteobacteria, and two to the Gammaproteobacteria.

### 3.4. Taxonomic Affiliations of phn Genes Under AEP Conditions

A total of 390 *phn* gene sequences were identified from the KEGG annotation results and were attributed to different taxonomic units according to the NCBI NR annotation results (Table 1 and Table S2). The results demonstrated that 97.7% of the *phn* gene sequences were attributed to Pseudomonadota. At the class level, 89.49% of *phn* gene sequences were affiliated with Alphaproteobacteria, indicating that Alphaproteobacteria are the primary group which possess the potential ability to utilize phosphonates.

An investigation into the distribution of *phn* gene sequences among the 20 most abundant genera revealed that ten of them, including *Labrenzia*, *Phycocomes*, *Pseudosulfitobacter*, *Aestuariivita*, *Mameliella*, *Limnobacter*, *Marivita*, *Roseobacter*, *Hoeflea* and *Roseovarius*, contain 12 to 14 of the 14 *phnC*~*phnP* genes, which are the encoding genes of the C-P lyase pathway (Table 2). Furthermore, the phosphonatase pathway genes *phnW* and *phnX* were identified in both *Labrenzia* and *Phycocomes*. Meanwhile, *phnW*, *phnY*, and *phnA*, which constitute another C-P hydrolytic pathway, were found in *Marivita*. Some *phn* genes, including *phnP*, *phnX*, and *phnA*, have been occasionally found in certain genera. However, these are not sufficient to form a complete phosphonate degradation pathway.

### 3.5. Isolation and Identification of Bacteria from AEP Condition

**Figure 4:**
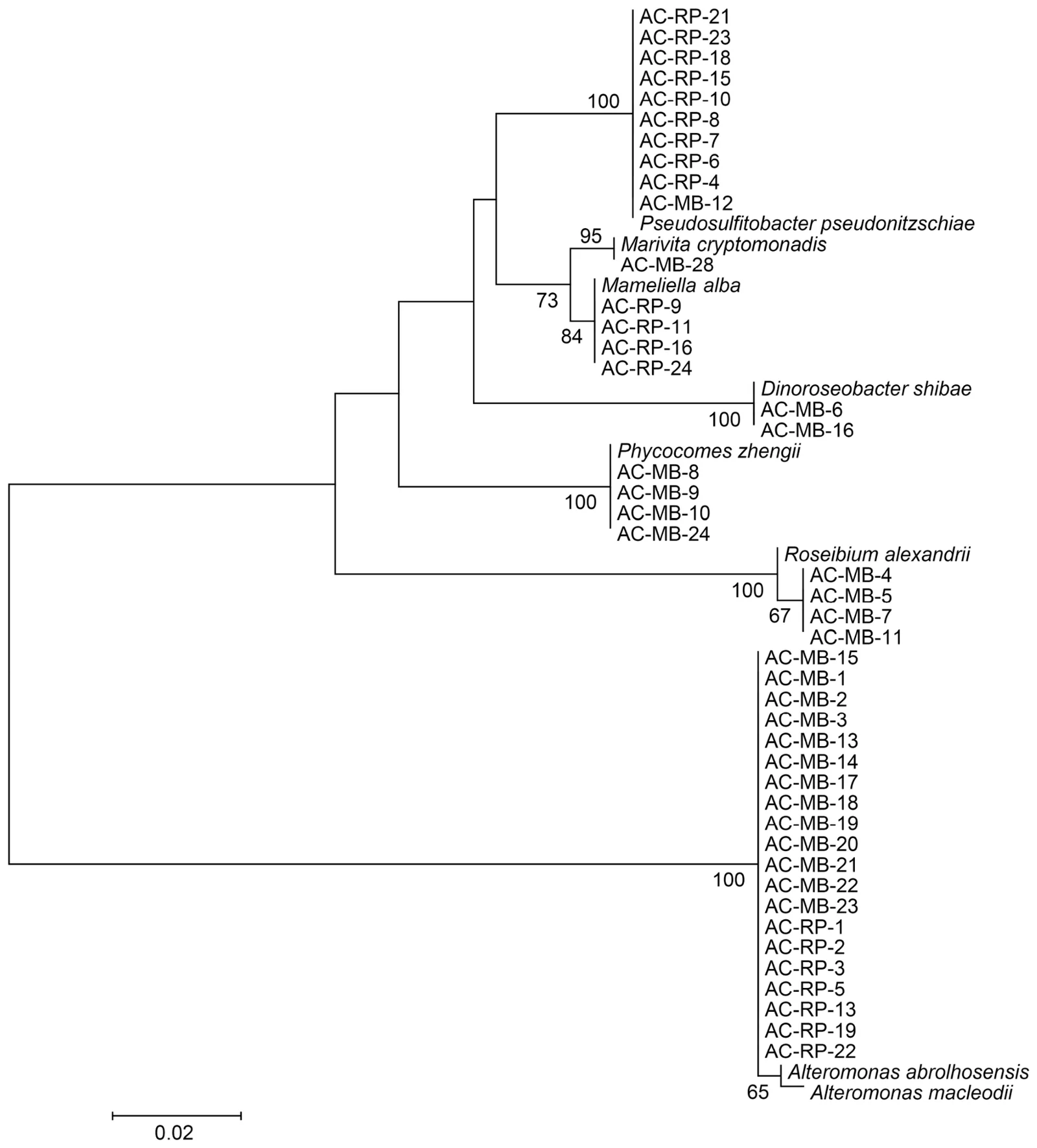
Neighbor-joining phylogenetic tree of 16S rRNA gene sequences from *Amphidinium carterae*-associated bacteria isolated in this study and their close relatives. Bootstrap values (percentages) based on 1000 replicates are shown at branch nodes (values < 50% are not shown).

In total, 25 and 20 bacterial strains were successfully isolated using MB and RP medium, respectively. BLAST analysis of their 16S rRNA gene sequences revealed that these strains were attributed to seven genera, including *Phycocomes*, *Pseudosulfitobacter*, *Mameliella*, *Roseibium*, *Marivita*, *Dinoroseobacter* and *Alteromonas*, with *Phycocomes zhengii* (currently *Meridianimarinicoccus zhengii*), *Pseudosulfitobacter pseudonitzschiae* (syn. *Sulfitobacter pseudonitzschiae*), *Mameliella alba*, *Roseibium alexandrii* (syn. *Labrenzia alexandrii*), *Marivita cryptomonadis*, *Dinoroseobacter shibae* and *Alteromonas abrolhosensis* (or *Alteromonas macleodii*) as their closest species (Table S3). The 16S rRNA gene sequence identity of these strains with their closest species is all above 98.7%, suggesting that they are likely the same species, which still requires further systematic verification. Bacterial strains assigned to six of the seven genera were isolated using MB medium, whereas only three genera were isolated via RP medium. The isolation of the *Mameliella* strains was only achieved in RP medium. The isolation of most of the *Pseudosulfitobacter* strains was derived from RP medium, with the exception of AC-MB-12. These results suggest that different bacterial groups exhibit a specific preference for distinct culture media. Phylogenetic analysis of the bacterial 16S rRNA gene sequences also demonstrated that all the isolated strains were clustered together with their closest relatives (Figure 4). Notably, the strains attributed to *Alteromonas* were clustered together with zero branch length, although BLAST analysis showed that some of them were close to *A. abrolhosensis*, while others were close to *A. macleodii*.

### 3.6. Distribution of phn Gene Clusters Among the Isolated Strains

Genomic analysis of type strains of *P. zhengii*, *P. pseudonitzschiae*, *M. alba*, *R. alexandrii*, *M. cryptomonadis*, *D. shibae,* and *A. abrolhosensis* and *A. macleodii* revealed that the genomes of the first five species all contain the *phn* genes that can form a complete phosphonate degradation pathway. A gene encoding a metal-dependent hydrolase involved in phosphonate metabolism was identified in the genome of *D. shibae* DFL12 (GenBank ID: GCA_000018145.1). *phnA* has also been identified in the genomes of *A. abrolhosensis* PEL67E (GenBank ID: GCA_001953635.1) and *A. macleodii* AD001 (GenBank ID: GCA_000808575.1), but these were the only identified *phn* genes in these genomes.

**Figure 5:**
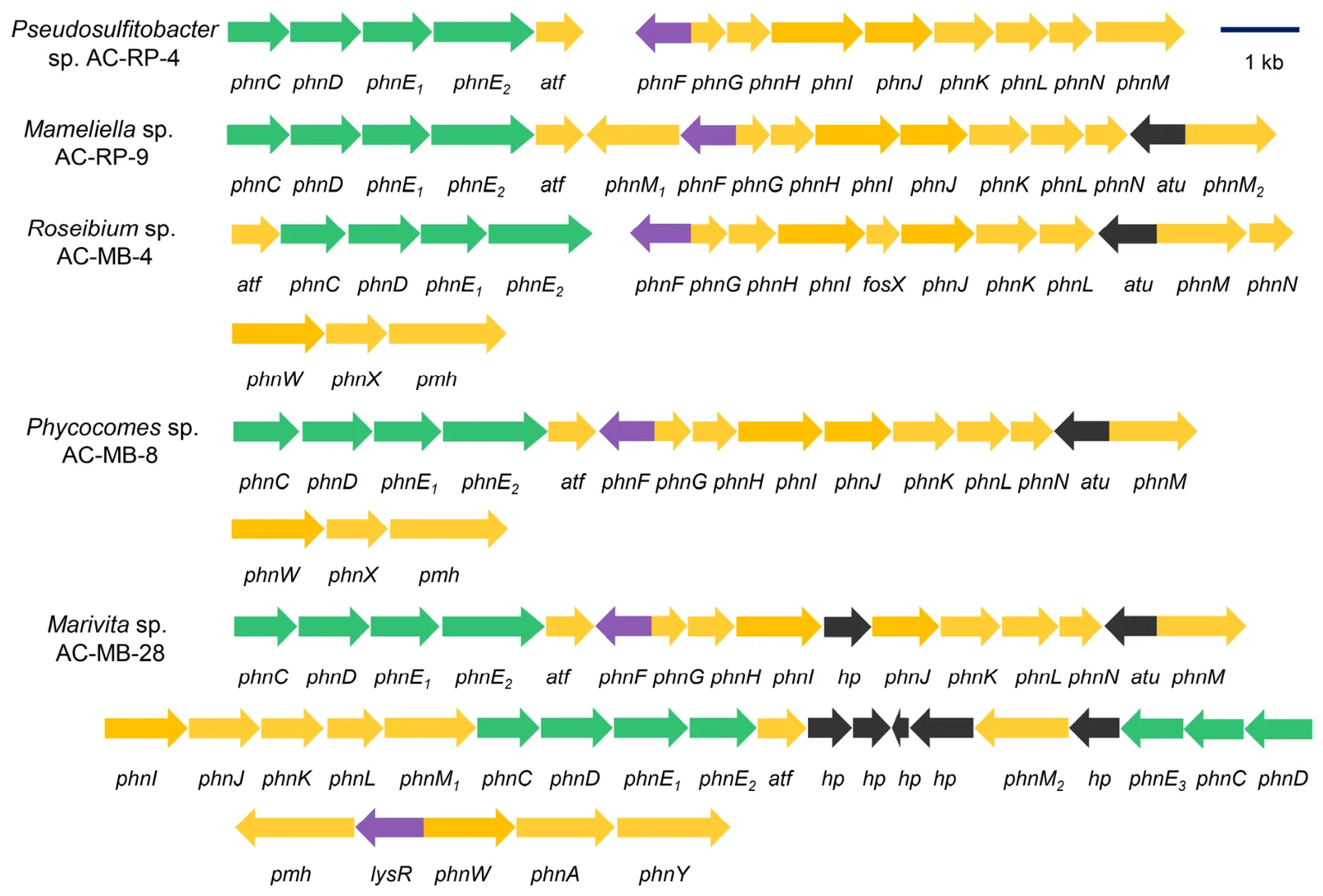
The organization of C-P lyase gene clusters in the genome of the five strains isolated in this study. Genes encoding phosphonate transport proteins (green), regulatory genes (purple), C-P lyase subunits (yellow), and other proteins of unknown function (black) are shown in different colors. *atf*: phosphonate utilization-associated acetyltransferase; *pmh*: phosphonate monoester hydrolase; *fosX*: fosfomycin resistance protein FosX; *lysR*: LysR-type transcriptional regulator; *atu*: uncharacterized protein Atu0170; *hp*: hypothetical protein.

The genomes of the representative strains from five genera of the isolated strains in this study, including *Pseudosulfitobacter* sp. AC-RP-4, *Mameliella* sp. AC-RP-9, *Roseibium* sp. AC-MB-4, *Phycocomes* sp. AC-MB-8, and *Marivita* sp. AC-MB-28 were sequenced and analyzed to ascertain the distribution of *phn* genes. The results indicate that all five strains possess genes that comprise one or more pathways associated with phosphonate degradation (Figure 5). Specifically, all five strains contain a gene cluster encoding the C-P lyase pathway, with AC-MB-28 harboring two C-P lyase gene clusters. The genes encoding the C-P lyase pathway in AC-RP-4 and AC-MB-4 are distributed in two separate clusters. The arrangement of genes encoding the C-P lyase pathway in these strains does not strictly follow the classic *phnC*-*phnP* order. It has also been observed that *phnO* and *phnP* are usually absent in their C-P lyase gene clusters. Conversely, *phnM* and *phnE* frequently occur in duplicate copies, and genes other than those of the *phnC*-*phnP* type, such as the genes encoding the phosphonate utilization associated acetyltransferase (Atf) and some hypothetical proteins, are also distributed across the same gene cluster. One of the hypothetical proteins was annotated as “uncharacterized protein Atu0170”, which was observed in the *phn* gene cluster of four bacterial strains. It is noteworthy that one gene coding for the fosfomycin resistance protein FosX was observed to exist in the *phn* gene cluster of AC-MB-4, suggesting its potential to resist the toxicity of fosfomycin, which is also a typical kind of biogenic phosphonate in nature.

Furthermore, analysis of these genomes revealed the presence of genes encoding other types of phosphonate metabolism pathways. The AC-MB-4 and AC-MB-8 strains contain the PhnW-PhnX pathway, while the AC-MB-28 strain possesses the PhnW-PhnY-PhnA pathway. *phnW* and *phnX* in the first two bacteria are located adjacent to *pmh* (encoding phosphonate monoester hydrolase). The genes responsible for encoding the PhnW-PhnY-PhnA pathway are also distributed in conjunction with other *phn* genes, including *pmh* and *lysR* (a transcriptional regulator from the LysR family).

### 3.7. Growth of Bacterial Strains Under Three Different Phosphorus Treatment Conditions

**Figure 6:**
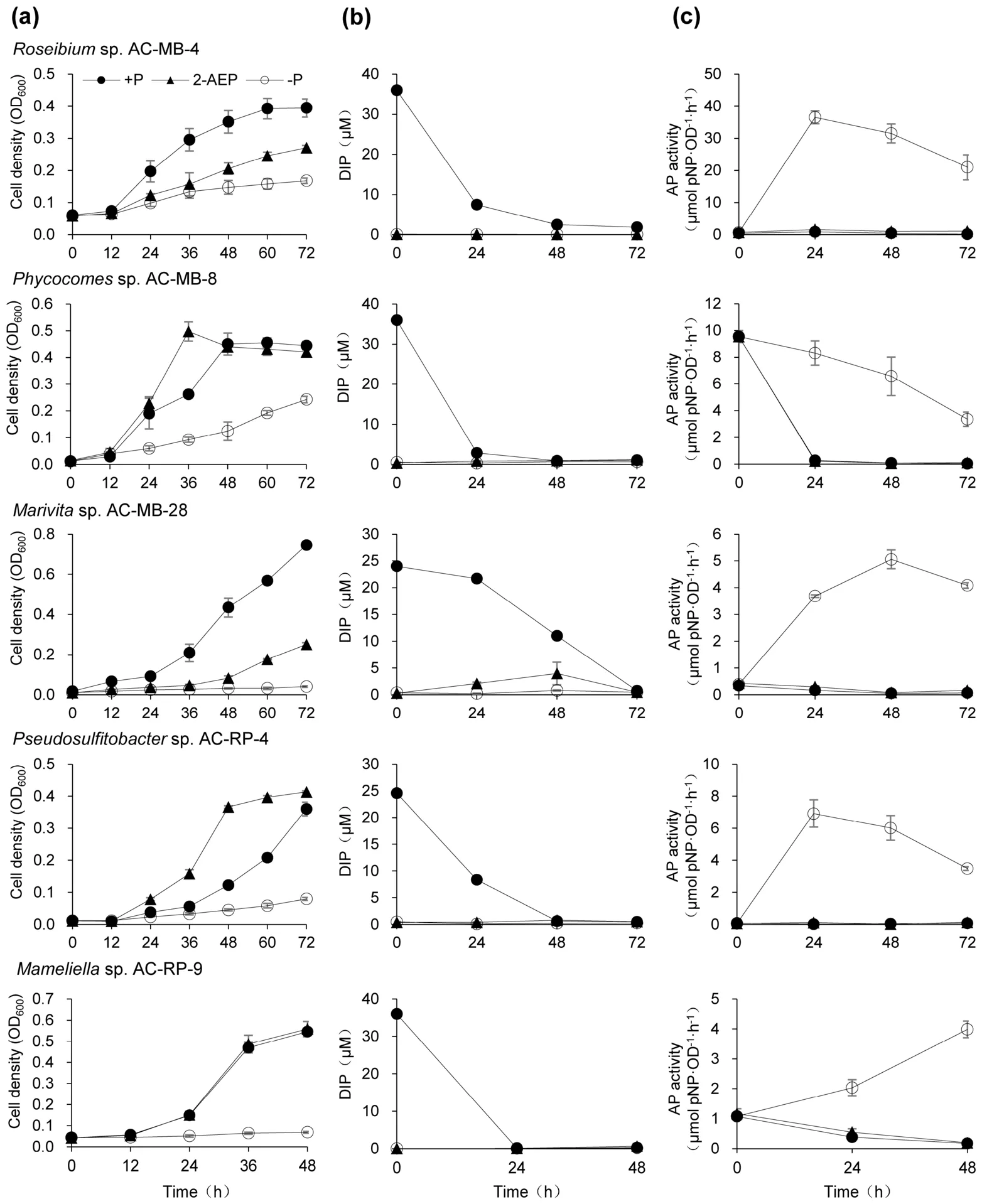
Effects of different phosphorus treatments on the growth and physiological responses of five bacterial strains. (**a**) Cell density; (**b**) dissolved inorganic phosphorus (DIP) concentration; (**c**) alkaline phosphatase (AP) activity. Cells were cultured under three phosphorus treatments: +P (36 μM NaH_2_PO_4_), AEP (36 μM 2-aminoethylphosphonate), and -P (no phosphorus added).

A series of culture experiments were conducted under three distinct P treatments to determine whether 2-AEP can support the growth of the five representative bacterial strains as the sole P source (Figure 6). The results demonstrate that there was a similar pattern in all five strains: in contrast to the -P condition, the bacterial cell density was found to be elevated at 48 or 72 h (*p* < 0.05) in the AEP condition. Conversely, AP activity level was found to be similar to that in the +P condition (*p* > 0.05) and significantly lower than the -P condition (*p* < 0.05). The DIP concentration in the AEP condition of most strains remained constant at a level comparable to that of the -P condition. However, the AC-MB-28 strain exhibited an exception, demonstrating a transient increase in DIP concentration under AEP conditions, as observed within the 24 to 48 h period, indicating that phosphate was released during this period. While the growth rates of these five representative strains were all higher under AEP conditions than under -P conditions, they exhibited different patterns when compared to +P conditions. For AC-MB-4, AC-MB-8, and AC-MB-28, the growth rate in the AEP condition was lower than the +P condition. AC-RP-9 exhibited a similar growth rate under conditions of 2-AEP and +P. However, AC-RP-4 demonstrated a higher rate of growth in the AEP condition than in the +P condition at 72 h.

## 4. Discussion

### 4.1. Bacterial Phosphonate Degradation Is Associated with Enhanced A. carterae Growth

Dinoflagellates constitute a significant group of marine phytoplankton and demonstrate a notable capacity to adapt to P-limited environments [1]. They can cope with P limitation through various mechanisms, including the utilization of DOP in the ocean [36,37]. However, previous studies have demonstrated that while numerous dinoflagellates possess genes associated with phosphonate metabolism, they lack the capacity to utilize extracellular phosphonates [19,38].

The results in our study demonstrated that the dinoflagellate *A. carterae* also cannot grow in antibiotic treatments with 2-AEP as the sole P source, indicating that it was incapable of utilizing 2-AEP as a P source to promote its growth (Figure 1a). This result is consistent with the findings of other researchers [22]. However, *A. carterae* demonstrated a distinct response when cultivated in a non-sterile system. Algal cell density and Fv/Fm under 2-AEP treatment conditions were significantly higher than under -P conditions, while AP activity was significantly decreased (Figure 2). The increase in AP activity has been established as an effective indicator of P deficiency in phytoplankton [39,40]. Meanwhile, Fv/Fm is a measure of the maximum light-to-energy conversion efficiency of phytoplankton and is typically reduced under conditions of nutrient stress, such as N or P deficiency [41,42]. Therefore, the above results indicate that, under AEP conditions, *A. carterae* cells that were previously in a P-starved state gained access to P, which helped them alleviate P limitation, promote their growth, and maintain relatively high photosynthetic efficiency. Since 2-AEP is the sole P source in the culture system, the P obtained by the algal cells most likely originates from the conversion of this phosphonate into a bioavailable form. Given that *A. carterae* cannot degrade 2-AEP independently, this process was likely mediated by bacteria in the culture system. This speculation was further supported by the filtrate experiment, where DIP concentrations increased markedly after 2-AEP addition to the algal cell-free filtrate (Figure 1b). Similar results have also been observed in other dinoflagellates, such as *P. donghaiense*, *Alexandrium pacificum*, and *Symbiodinium kawagutii* [19].

Furthermore, algal cell density and Fv/Fm under the 2-AEP treatment were lower compared to the +P treatment, while APA was higher. This suggests that algal cells still faced P limitation to some degree under these conditions. This finding suggests that the rate of bacterial degradation of 2-AEP is inadequate to fully satisfy the demand of algal cells for P. DIP levels remained at extremely low levels under AEP conditions, suggesting that the phosphate produced by bacterial degradation had been rapidly absorbed. Through phosphonate degradation, bacteria can obtain not only P but also carbon (C) or N [43,44]. This process enables bacteria to release excess P into the extracellular environment. This may also explain the increase in DIP concentration observed in the FL+2-AEP condition (Figure 1b).

It is important to note that bacteria also competitively absorb this released phosphate. However, the concurrent increase in algal cell density, Fv/Fm, and reduced AP activity collectively indicate that the algal cells did benefit from an enhanced P supply. Taken together, despite the absence of direct evidence for P transfer from bacteria to algae, the growth promotion is most likely due to bacterial degradation of phosphonates. Future studies employing ^33^P-labeled 2-AEP could directly trace the flux of P from bacterial degradation to algal uptake, thereby providing definitive evidence for this proposed bacterial–algal P transfer.

### 4.2. Diversity of Phosphonate-Degrading Bacteria and Degradation Pathways in A. carterae-Associated Bacteria

The bacterial community structure analysis revealed that Pseudomonadota was the dominant phylum in the *A. carterae*-associated community under 2-AEP treatment, with Alphaproteobacteria as the most dominant class (Figure 3). This result is generally consistent with previous studies, except for the overwhelming dominance of this phylum. Pseudomonadota and Bacteroidota have been documented as the dominant phyla within the bacterial community in cultures of *P. donghaiense*, with Bacteroidetes exhibiting a particularly high percentage in routine f/2 cultures [21]. A previous study has revealed that Alphaproteobacteria, Gammaproteobacteria, and the Flavobacteria–Sphingobacteria group within the Bacteroidota phylum constitute the predominant group in seven freshly isolated key diatom and dinoflagellate species [45].

However, the composition of the bacterial community in the microalgal culture may be influenced by numerous factors, including the type of microalgal species, the isolation source of the algae, the duration of their maintenance in the laboratory, and the culture conditions under which they are maintained [46]. Accordingly, the bacterial community associated with the laboratory-maintained *A. carterae* strain used in this study may not fully reflect that of natural phycosphere assemblages. Studies have demonstrated that the supplementation of different P forms exerts a substantial impact on the composition of bacterial communities within aquatic environments [47]. However, whether the bacterial community structure was influenced by the presence of 2-AEP remains to be investigated in future studies. Nevertheless, it is possible that bacteria capable of phosphonate degradation were favored under the 2-AEP condition.

The distribution of the *phn* genes at the genus level shows that genes found in 10 genera—including *Labrenzia*, *Phycocomes*, *Pseudosulfitobacter*, *Aestuariivita*, *Mameliella*, *Roseovarius*, *Limnobacter*, *Marivita*, *Roseobacter*, and *Hoeflea*—have the potential to constitute a complete phosphonate degradation pathway (Table 2). They all contain most of the major genes that make up the C-P lyase pathway. Furthermore, the genera *Labrenzia* and *Phycocomes* both contain the two genes encoding the complete PhnW-PhnX pathway, while the genus *Marivita* contains the three genes encoding the complete PhnW-PhnY-PhnA pathway. Nine of these 10 genera are classified under the Alphaproteobacteria, while *Limnobacter* belongs to the Betaproteobacteria. This result is aligned with the distribution pattern of the *phn* gene at the class level (Table 1).

Bacterial strains belonging to seven genera were isolated from the AEP condition. It is highly probable that they represent seven distinct species (Figure 4). AC-MB-1, AC-MB-4, AC-MB-6, AC-MB-8, AC-MB-28, AC-RP-4, and AC-RP-9 were selected as representative strains for further study. They exhibited the closest phylogenetic affinity to *A. abrolhosensis*, *R. alexandrii*, *D. shibae*, *P. zhengii*, *M. cryptomonadis*, *P. pseudonitzschiae,* and *M. alba*, respectively. A close association between the above species and dinoflagellates is supported by the isolation of five of them from this particular phytoplankton group [48,49,50,51,52]. *P. zhengii* and *P. pseudonitzschiae* have been isolated from green algae (*Chlorella vulgaris*) and the diatom (*Skeletonema marinoi*), respectively, indicating that they are also typical microalgal-associated bacteria [53,54].

Genomic analysis of AC-MB-4, AC-MB-8, AC-MB-28, AC-RP-4, and AC-RP-9 reveals the presence of the phosphonate degradation pathway in the genomes of all five bacterial strains (Figure 5). However, neither of the model bacterial species *A. abrolhosensis* nor *D. shibae* contains a phosphonate degradation pathway, indicating that AC-MB-1 and AC-MB-6 likely lack the genetic potential to degrade phosphonates. This indicates that the former five bacterial strains have the potential to utilize phosphonates.

In summary, the metagenomic and genomic analysis results from this study reveal a rich species diversity of phosphonate-degrading bacteria among *A. carterae*-associated bacteria. Furthermore, these bacteria demonstrate a diverse variety of phosphate degradation pathways, encompassing the C-P cleavage enzyme pathway and two C-P hydrolase pathways: the PhnW-PhnX pathway and the PhnW-PhnY-PhnA pathway.

The C-P lyase pathway was first identified in *Escherichia coli*, where 14 genes—including *phnC* through *phnP*—are arranged sequentially in its genome [55]. In contrast, the composition and arrangement of the *phn* genes in the C-P lyase pathway among the five bacterial species isolated in this study (Figure 5) exhibited several distinct characteristics: (1) the absence of *phnO* and *phnP*, as well as the presence of genes other than *phnC* to *phnP*, such as *fosX* and some genes of unknown function; (2) the arrangement of the *phn* genes does not strictly adhere to the *phnC*–*phnN* order; (3) more than one *phnE* or *phnM* gene is present within the same C-P lyase gene cluster; and (4) in certain bacterial species, the genes encoding the C-P lyase pathway are not located within the same gene cluster, as has been observed in AC-RP-4 and AC-MB-4. The gene rearrangements and multiple copies observed in these *phn* gene clusters may enhance the functional flexibility of the bacterial community, potentially enabling the utilization of diverse phosphonate substrates in the marine environment.

Furthermore, AC-MB-28 contains two independent C-P lyase pathways, a phenomenon that has also been observed in *Pseudomonas stutzeri* [56]. In addition, AC-MB-28 also contains the PhnW-PhnY-PhnA pathway. AC-MB-4 and AC-MB-8 also include other phosphonate degradation pathways besides the C-P lyase pathway, which is the PhnW-PhnX pathway. Gene clusters encoding both pathways identified in this study contain a *pmh* gene, which encodes the phosphonate monoester hydrolase, a metal-dependent enzyme that catalyzes the hydrolysis of phosphonate monoesters [57]. The PhnW-PhnY-PhnA cluster also contains a *lysR* gene, which encodes a LysR-type transcriptional regulator, suggesting its potential role in regulating the pathway [58].

### 4.3. Multiple Degradation Pathways Potentially Enhance Phosphonate Utilization and Algal Growth Promotion

The results of the bacterial culture experiments demonstrated that, in comparison to the -P conditions, the density of the five examined bacterial strains increased, while AP activity decreased under AEP conditions, indicating that all of them are capable of degrading 2-AEP (Figure 6). Given the limited number of strains that can be isolated and cultured in a laboratory environment, it is presumed that the *A. carterae*-associated bacterial community likely contains a highly diverse array of phosphonate-degrading bacteria. This speculation is further supported by the identification of complete phosphonate degradation pathways in 50% of the 20 most abundant genera within this community (Table 2). These phosphonate-degrading bacteria have the potential to facilitate the growth of algal cells in environments with limited phosphate availability by breaking down phosphonates into phosphate, which is subsequently absorbed by the algal cells. This mechanism may even play a role in the maintenance of algal blooms, as has also been investigated in the bloom-forming cyanobacterium *Microcystis* [59].

Among the bacterial phosphonate degradation pathways, the C-P lyase pathway is typically subject to regulation by the Pho regulon, and its expression is inhibited by phosphate [11,60]. However, the expression of the PhnW-PhnX and PhnW-PhnY-PhnA pathways is generally considered to be substrate-induced and phosphate-independent. The presence of phosphate-unlimited 2-AEP degradation pathways within the dinoflagellate-associated bacterial communities is supported by the observations from both this study (Figure 1b) and previous studies [19], in which phosphate concentration increased steadily in filtrates of dinoflagellate cultures when 2-AEP was supplied as the sole P source.

It is hypothesized that the three pathways operate in a complementary pattern to sustain phosphonate degradation across a range of ambient phosphate concentrations, based on these distinct regulatory properties. In circumstances where phosphate levels are negligible, the C-P lyase pathway could be induced, which would enable a rapid response to the existence of phosphonate substrates, such as 2-AEP. At the same time, 2-AEP can also trigger the expression of two other signaling pathways. Phosphonate degradation might temporarily increase phosphate concentration in ambient water, inhibiting the C-P lyase pathway, but the other two pathways continue to function. Meanwhile, phosphate present in the water is typically rapidly absorbed by dinoflagellates, both in natural aquatic environments and in laboratory-based algae–bacteria co-culture experiments. This rapid removal of phosphate inhibition enables the continuous degradation of phosphonates through the C-P lyase pathway.

In summary, dinoflagellate-associated bacteria possess the capacity to degrade phosphonates via multiple pathways, regardless of the ambient phosphate concentration. The phosphonate utilization by these bacteria could potentially provide P to their host dinoflagellates, especially for those bacterial groups that acquire not only P but also C or N. Conversely, the bacteria may also benefit from the growth of the dinoflagellates through the acquisition of photosynthetic products from their algal host. This symbiotic mechanism could not only enhance dinoflagellate resilience to phosphate limitation but may also contribute to bloom development by supplying an alternative P source. The phosphonate degradation by the bacteria associated with dinoflagellates and other phytoplankton groups also allows this recalcitrant organic P to be indirectly utilized by the primary producers, thereby also performing a certain role in the marine P cycle.

## 5. Conclusions

The present study investigated the phosphonate-degrading groups among bacteria associated with the dinoflagellate *A. carterae* and the phosphonate degradation pathways within these groups. The results indicated that this bacterial community exhibits remarkable biodiversity, as well as considerable diversity in terms of phosphonate degradation pathways. Among the 20 most abundant genera in the samples, 10 possess the genetic potential to degrade phosphonates, with the majority belonging to the Alphaproteobacteria. Phosphonate metabolism can be performed through multiple routes, including the C-P lyase pathway, the PhnW-PhnX pathway, and the PhnW-PhnY-PhnA pathway. This metabolic diversity enhances the persistence of this function across varying phosphate concentrations. Five bacterial strains from different genera were isolated and confirmed to be capable of utilizing 2-AEP, providing a basis for future studies on their molecular regulatory mechanisms and roles in dinoflagellate-bacteria interactions. These phosphonate-degrading bacteria have great potential to serve as a microbial strategy that supports dinoflagellate adaptation to P limitation. This symbiotic mechanism sheds new light on algae–bacteria dynamics while also serving as an important driver of biogeochemical P cycling. Further investigations are necessary to thoroughly examine this ecologically relevant process.

## Figures and Tables

**Table 1:** Taxonomic assignment of the 390 identified *phn* gene sequences at the phylum and class level.

Phylum	Class	*phn* Gene Number	Proportion (%)
Pseudomonadota	Alphaproteobacteria	349	89.49%
	Betaproteobacteria	26	6.67%
	Gammaproteobacteria	6	1.54%
Bacteroidota	Saprospiria	4	1.03%
	Flavobacteriia	3	0.77%
Actinomycetota	Actinomycetes	1	0.26%
Cyanobacteriota	unclassified class of Cyanobacteriota	1	0.26%

**Table 2:** Relative abundances of major bacterial genera under AEP treatments and the distribution of their associated phosphonate metabolism genes across different pathways.

Genus	Relative Abundance	C-P Lyase Pathway	PhnW-PhnX Pathway	PhnW-PhnY-PhnA Pathway
Labrenzia *	22.52%	***phnC~P***	***phnW, phnX***	-
Phycocomes *	17.38%	***phnC~N, phnP***	***phnW, phnX***	-
Pseudosulfitobacter *	10.15%	***phnC~N, phnP***	-	*phnA*
*Roseibium*	6.82%	*phnD*	-	-
Aestuariivita *	4.70%	***phnC~N, phnP***	-	-
Mameliella *	4.48%	***phnC~J, phnL~N, phnP***	-	*phnA*
*Oceanicaulis*	3.70%	*phnP*	-	-
Roseovarius *	3.26%	***phnC~F, phnH, phnI, phnK~N, phnP***	-	-
Limnobacter *	2.98%	***phnC~N, phnP***	-	-
*Candidatus Phaeomarinobacter*	2.82%	*phnP*	-	-
*Dinoroseobacter*	2.51%	*phnC~E, phnP, phnM*	-	-
*Phaeodactylibacter*	2.41%	-	*phnX*	*phnA*
Marivita *	1.88%	***phnC~P***	-	***phnW, phnY, phnA***
*Methylophaga*	1.27%	*phnC~E*	-	*phnA*
Roseobacter *	0.86%	***phnC, phnE, phnG~J, phnL, phnN***	-	*phnY*
*Marinicauda*	0.79%	-	-	-
*Sulfitobacter*	0.75%	*phnC*	-	-
Hoeflea *	0.58%	***phnC~J, phnL~N, phnP***	-	*phnA*
*Alkalicaulis*	0.50%	-	-	-
*Alteromonas*	0.21%	*phnD*	-	*phnA*

Note: An asterisk next to a genus name (in bold) denotes a genus with a complete phosphonate degradation pathway, and gene names in bold indicate the genes involved in the complete pathway. Complete pathways were defined as follows: C-P lyase, presence of most of the core genes *phnG*~*phnM*; PhnW-PhnX, presence of both *phnW* and *phnX*; PhnW-PhnY-PhnA, presence of *phnW*, *phnY*, and *phnA*. “-” indicates that no corresponding gene was identified.

## Data Availability

The original contributions presented in this study are included in the article/Supplementary Material. Further inquiries can be directed to the corresponding author.

## Supplementary Materials

The following supporting information can be downloaded at https://www.mdpi.com/article/10.3390/microorganisms14092028/s1. Table S1: Summary of sequencing and assembly statistics for metagenomic samples from the AEP treatment; Table S2: *phn* gene sequences identified from metagenomic analysis with taxonomic and functional annotations; Table S3: Bacterial strains isolated from *Amphidinium carterae* on MB and RP medium and their 16S rRNA gene BLAST results.
